# Postoperative Hemodynamics of Total Knee Arthroplasty Unaffected by Cementless Approach under Contemporary Patient Blood Management Protocol: A Propensity Score-Matched Study

**DOI:** 10.3390/jcm12226980

**Published:** 2023-11-08

**Authors:** Keun Young Choi, Yong Deok Kim, Nicole Cho, Man Soo Kim, Yong In, Hwang Yong You, In Jun Koh

**Affiliations:** 1 Department of Orthopaedic Surgery, Seoul St. Mary’s Hospital, Seoul 06591, Republic of Korea; heaxagon@hanmail.net (K.Y.C.); kms3779@naver.com (M.S.K.); iy1000@catholic.ac.kr (Y.I.); 2 Department of Orthopaedic Surgery, College of Medicine, The Catholic University of Korea, Seoul 06591, Republic of Korea; seraph622@naver.com (Y.D.K.); yhy30519@cmcnu.or.kr (H.Y.Y.); 3 Joint Replacement Center, Eunpyeong St. Mary’s Hospital, Seoul 03312, Republic of Korea; 4 Lauren E. Wiznia MD PLLC, 1016 Fifth Avenue, New York, NY 10028, USA; ncho1210@gmail.com

**Keywords:** total knee arthroplasty, cementless prosthesis, patient blood management, propensity score matching, hemodynamics

## Abstract

(1) Background: A cementless total knee arthroplasty (TKA) is a recent and an increasingly popular innovation that enhances porous fixation surfaces. However, the lack of cemented sealing of an exposed resected bone has raised concerns about the potential for greater blood loss. The goals of this study were to determine if a cementless approach impacts post-TKA hemodynamics and to identify risk factors for blood loss in instances of cementless (vs. cemented) TKAs under a contemporary patient blood management (PBM) protocol. (2) Methods: We recruited 153 consecutive patients undergoing unilateral TKAs between 2019 and 2023. All enrollees received cementless or cemented prostheses of the same design (cementless, 87; cemented, 66). After propensity score matching for demographics, there were 46 patients in each group. We then compared blood loss metrics (total [TBL] and estimated [EBL]), drainage volumes, hemoglobin (Hb) levels, and transfusion rates by group. (3) Results: Post-TKA hemodynamics (i.e., TBL, EBL, drainage, Hb level, and transfusion rate) of cementless (n = 46) and cemented (n = 46) TKA groups did not differ significantly. In addition, the proportions of patients with Hb drops > 3.0 g/dL were similar for the two groups. A logistic regression analysis revealed that only preoperative Hb and EBL during the early postoperative period were predictive of a substantial fall in Hb levels. The fixation method was not associated with Hb decline > 3.0 g/dL by postoperative Day 3. (4) Conclusion: The cementless TKA has no impact on customary post-TKA hemodynamics and is not associated with greater TKA-related blood loss when implementing a contemporary PBM protocol.

## 1. Introduction

**Figure 1:**
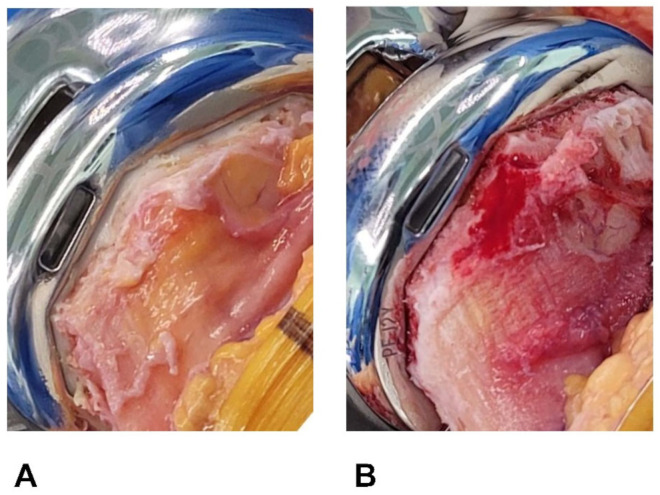
(**A**,**B**) Resected bone surfaces after implant fixation. (**A**) Bone cut surface is covered in cement after cemented TKA. (**B**) Resected surface of cancellous bone is exposed following cementless TKA.

There is a notable trend change in the demographic field of lower-extremity arthroplasty. Particularly, younger and more active patients who undergo total knee arthroplasties (TKAs) are witnessing a decline in the 10-year implant survival rates compared to older recipients, and they are rapidly increasing in number [1,2,3,4,5]. Consequently, biologic fixation through a cementless TKA has regained popularity [6,7,8]. However, this leaves resected surfaces of a cancellous bone exposed, and no cement layers offer a tamponade effect (Figure 1). Without such sealing, some investigators have reported greater blood loss after cementless (vs. cemented) TKAs [9,10], whereas others have shown comparable blood loss for the two procedures [11,12]. Whether cementless TKA actually promotes postsurgical blood loss to increase transfusion rates remains an unsettled matter.

Patient blood management (PBM) protocols have recently gained prominence as innovative measures to mitigate postoperative blood loss and transfusions. Although PBM is not defined by a single protocol and can be implemented in various modes depending on the institutional context and the surgeon’s experience, these protocols have garnered widespread acceptance in the medical community, finding application across a multitude of surgical disciplines. Notably, they have yielded significant reductions in post-total knee arthroplasty (TKA) blood loss and transfusion rates when implemented, as indicated by several studies [13,14]. Postoperative anemia is a presumed contributor to diminished quality of life and poor clinical outcomes [15,16], at times prompting life-threatening complications [17]. In fact, allogenic blood transfusion (the traditional remedy for postoperative anemia) may ultimately encourage patient morbidities, including periprosthetic joint infection (PJI) [18]. Current PBM protocols are known to improve postoperative hemoglobin (Hb) levels [9,13] and have benefitted lower-extremity arthroplasties by reducing allogenic blood transfusions and readmission rates [13,14]. Nonetheless, the impact of contemporary PBM on post-TKA hemodynamics remains unclear with respect to present-day cementless TKA.

This propensity score-matched study was conducted to determine whether a cementless TKA affects post-TKA hemodynamics, namely, blood loss, Hb levels, and transfusion rates. We also sought to identify risk factors for greater blood loss in patients undergoing a TKA under the current PBM protocol. Our perspective was that a cementless TKA would not affect post-TKA hemodynamics and that the choice of fixation method would not be a factor in patient blood loss. We hypothesized that blood loss in a cementless TKA would be similar to that of the cemented counterpart and that hemodynamic variables including transfusion rate and perioperative surgical variables would be similar to a shorter operation time in the cementless group in unilateral TKAs using the propensity score matching (PSM) method and that the mode of fixation of a TKA prosthesis is not associated with a higher blood loss after unilateral TKAs under the current updated PBM protocols.

## 2. Materials and Methods

**Figure 2:**
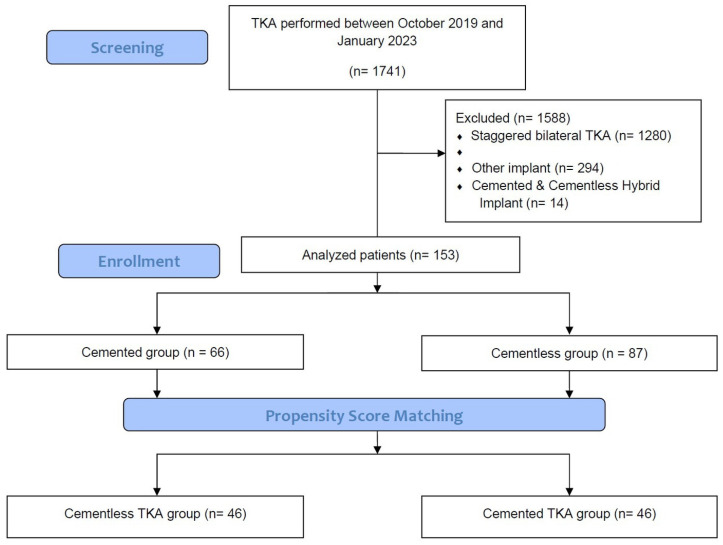
Flow diagram.

Between October 2019 and January 2023, a total of 153 patients underwent unilateral TKAs (cementless, 87; cemented, 66). Eligible patients were older than 20 years and undergoing unilateral TKAs with cementless or cemented implants with a minimum follow-up interval of 3 months because of primary knee osteoarthritis (OA). The study protocol excluded patients with a history of prior knee surgery on the knee, pre-TKA hemoglobin (Hb) less than 10 g/dL, a diagnosis of inflammatory arthritis or secondary arthritis, prior fracture of the knee, categorized ASA grade IV or higher, or severe limitation of motion (a flexion contracture greater than 25° or further flexion less than 90°). Use of cementless TKA was dependent on intraoperative bone quality, resulting in significant group-wise demographic heterogeneity. This was rectified through PSM, creating two groups (n = 46 each) based on age, sex, and body mass index (BMI) (Figure 2). In general, a propensity score refers to the conditional probability of a specific treatment being administered to an individual with a given set of characteristics [19,20]. Observational studies frequently contend with the challenge of bias stemming from substantial dissimilarities between subjects in the treatment and no-treatment groups. These disparities can confound the interpretation of study results and undermine the validity of the findings. Recognizing the need to enhance the robustness of our study, we strategically employed the Propensity Score Matching (PSM) analysis as a method to counteract potential confounding variables [19,20]. The cementless and cemented groups were propensity-score-matched in a 1:1 ratio using a balanced, nearest-neighbor method. The study design was approved by the institutional review board of our hospital (PC23RISI0114). Except for height, there were no significant demographic differences between groups after PSM (Table 1).

All TKAs were assigned and performed by the same senior surgeon (one of the authors) using cementless (Triathlon Tritanium; Stryker Corp, Kalamazoo, MI, USA) or cemented (Triathlon; Stryker Corp) prostheses. The fixation method was determined based on intraoperative bone quality at the discretion of the operator. With a pneumatic tourniquet in place (at 250 mmHg), we followed the medial parapatellar approach, cutting the distal femur (intramedullary guide) and tibia (extramedullary guide). For cementless TKA, four additional peg holes were made on the tibial cut surfaces to accommodate four cruciform-shaped base-plate pegs. The femoral and tibial prostheses were otherwise fixed with two packs of bone cement (Doujet; Injecta, Seoul, Republic of Korea) using a vacuum mixing system and a one-stage cementation method. Once the real prosthesis and the polyethylene (PE) insert were installed, we deflated the tourniquet, followed by thorough bleeding control with a coagulator. Povidone-iodine and saline soaks were also applied for 3 min to prevent PJI. Capsular and subcutaneous layers were subsequently closed, and skin closure was achieved through topical adhesive and noninvasive Zip devices (Stryker Corp).

The same perioperative management protocol was followed for both procedures. On days of surgery, patients were advised to extend their knees hourly in sitting positions. Ambulation using a walker was then encouraged on postoperative Day 1, and active range-of-motion exercises were started. We discharged patients on postoperative Week 1, scheduling follow-up visits for Weeks 2 and 6, Months 3 and 6, Year 1, and yearly thereafter. This consistent approach ensures that patients receive the necessary and identical care and support at various stages of their recovery journey, excluding any factors arising from different postoperative management protocols.

Contemporary PBM protocols were extended to all patients (without exception), administering systemic and topical formulations of tranexamic acid (TXA). We slowly delivered intravenous (IV) TXA (500 mg) in normal saline (100 mL) 1 h after the skin incision and then 1 h again postoperatively in the hospital ward. We also used topical TXA (1 g) in normal saline (50 mL) to infuse the capsule and surrounding soft tissue prior to repair, clamping indwelling intra-articular catheters during the first 6 h after TKA and removing them within 24 h. Oxygen was administered via nasal prongs at a rate of 2 L/minute for 24 h postoperatively. Patients with Hb levels of <10 g/dL received IV ferric carboxymaltose (FCM), Ferinject^®^ (Vifor Pharma, Flughofstrasse, Switzerland), except for those who refused to get infusion. The Ferinject^®^ infusion was administered, as has been previously described: bodyweight 50 kg; 1000 mg of Ferinject^®^ mixed with 200 mL normal saline, bodyweight < 50 kg; 500 mg of Ferinject^®^ mixed with 100 mL normal saline [21,22]. Patients with an Hb level of <10 g/dL and a serum ferritin level of <15 ng/mL after postoperative 4 weeks were planned to receive an additional dose of 500 mg of FCM [23]. Finally, the transfusion threshold was set at a hemoglobin level of <7.0 g/dL within 3 days after surgery. However, patients with anemic symptoms or underlying cardiovascular disease were transfused at <8.0 g/dL. Aspirin and intermittent pneumatic compression were provided to all patients to prevent venous thromboembolism, and high-risk patients received additional intravenous low-molecular-weight heparin (enoxaparin). During procedures, we aimed for gentle and minimal soft tissue manipulation and brevity of operative times.

The primary outcome measure was estimated blood loss (EBL), calculated using the Lopez-Picado formula [24] as follows:

$$\frac{Patient\;blood\;volume\;(PBV)\;\times \;(preoperative\;Hct\;-\;postoperative\;[Day\;1\;or\;3]\;Hct)}{mean\;Hct\;(above\;time\;frame)}$$

PBV was calculated using the International Council for Standardization in Haematology (ICSH) formula [25]. Secondary outcomes included transfusion rates, Hb and hematocrit (Hct) levels, and proportion of patients with Hb drops > 3 g/dL.

### Statistical Analysis

In comparing primary and secondary group outcomes, independent *t*-tests and Chi-squared tests served to analyze continuous and categorical variables, respectively. To identify risk factors for greater TKA-related blood loss under contemporary PBM, patients were grouped as substantial vs. average Hb decline using a cut point of 3.0 g/dL on postoperative Day 3. This figure reflects the average Hb drop recorded after TKA [26]. We conducted simple and multiple logistic regression analyses in a backward stepwise manner for variables with a low *p*-value in the univariate analyses, generating odds ratios (ORs) and 95% confidence intervals (CIs). A power analysis indicated that 80% power was required to detect a 250 mL difference in procedural TBLs through two-sided testing at a significance level of 0.05. Statistical analyses were performed using SPSS (IBM Corp, Armonk, NY, USA) setting significance at *p* < 0.05.

## 3. Results

**Figure 3:**
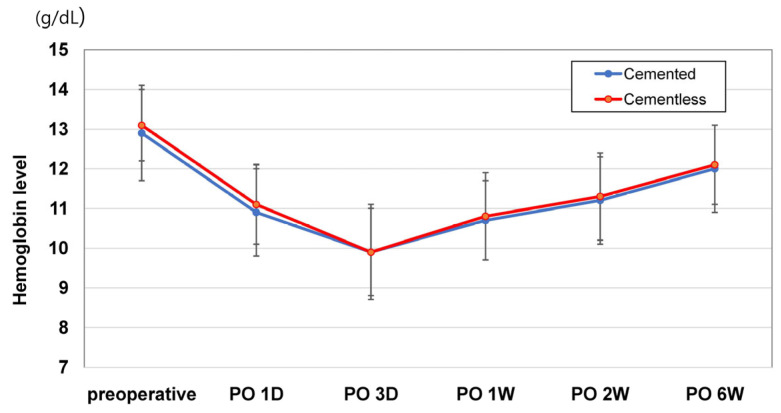
Changes in postoperative hemoglobin levels during the postoperative 6-week period. There were no differences in post-TKA Hb levels between cemented and cementless TKAs in all comparisons.

Each patient in both study groups received follow-up care exceeding a three-month duration, ensuring comprehensive postoperative monitoring and assessment for perioperative hemodynamic variables. On postoperative Days 1 and 3, EBL levels showed no significant group differences. Likewise, total intraoperative blood loss volumes did not differ; and because no patient in either group met transfusion criteria, transfusion rates also proved similar. The proportions of patients in each group with substantial Hb declines (>3 g/dL) on postoperative Days 1 and 3 and at postoperative Weeks 1, 2, and 6 showed no differences after PSM (all *p* > 0.05) (Table 2). Changes in postoperative Hb levels at all time points in the 6-week postoperative period were not significantly different for the two groups (Figure 3). Our cementless (vs. cemented) group registered shorter tourniquet (after PSM: 24.3 ± 6.9 min vs. 29.7 ± 7.6 min; *p* < 0.001) times before and after PSM (Table 1).

A simple logistic regression analysis revealed that prosthesis fixation, whether cemented or not, failed to impact Hb change >3.0 g/dL on postoperative Day 3 (*p* = 0.464). Preoperative Hb values and EBL levels on postoperative Days 1 and 3 were significantly higher in the group with substantial Hb decline (Table 3), but no other demographic, medical, or surgical parameters emerged as risk factors for substantially reduced Hb (all *p* > 0.05). Multiple logistic regression analyses performed with four predictors (fixation method, hypertension, EBL levels on postoperative Day 3, and type of anesthesia) revealed that EBL on postoperative Day 3 was the only significant risk factor for a substantial Hb drop (OR: 1.006, 95% CI: 1.004–1.008, *p* < 0.001).

## 4. Discussion

The number of younger and more active patients who require biological prosthesis fixation for TKA implant longevity is currently increasing [24]. Unlike the first-generation cementless prosthesis which failed because of multiple causes including flows of design such as metal-backed patella, recently cultivated cementless procedures are now considered successful substitutes for cemented counterparts in terms of clinical outcomes, displaying shorter operative times by comparison [11]. However, the issue of blood loss, presumably due to a lack of a sealing effect on resected bone, has yet to be resolved [9,10,11,12]. To our knowledge, there have been no observational studies such as ours comparing cementless and cemented TKAs under a strict PBM protocol, while excluding confounding factors, through PSM.

The most important finding herein is that the post-TKA hemodynamics of the two matched groups did not differ significantly, with less tourniquet and operative times recorded for cementless procedures. Our data align with prior studies reporting similar EBL levels for both groups [11,12] and supporting the assertion that a cementless TKA does not affect post-TKA hemodynamics. Successful clinical outcomes and implant survival have already been well-documented in recent accounts of cementless TKAs [10,12,25], as have shorter operative and tourniquet times [26]. The duration of a TKA is even cited as one of the key factors in developing postoperative infection [27]. Despite the absence of PJI in our cohort, the sampling size (N = 153) was relatively small. In a TKA procedure lasting about 1 h, a time reduction of 5 min may perhaps help to mitigate such risk. While a larger study is needed to validate this, based on our observations, a cementless TKA is a valid substitute for cemented procedures.

In previous studies, some researchers have determined a greater blood loss for cementless (vs. cemented) TKAs [9,10], a finding we must challenge. The inconsistencies and controversies over TKA-related blood loss are possibly rooted in the variety of PBM protocols intended for an arthroplasty, rather than the method of prothesis fixation. More recently, PBM has been applied to an array of surgical procedures in hopes of improving patient outcomes and facilitating required blood transfusions [28]. Although now generally accepted by many surgical departments, there is no universal PBM protocol to date [29]. Nonetheless, reports of reduced blood loss, lower transfusion rates, and better Hb recovery after a TKA continue to accumulate [3,13,29,30,31,32]. Recently, the most representative PBM protocol incorporates hypotensive anesthesia, drain clamping, application of pneumatic tourniquets, strict transfusion triggers, IV or topical administration of TXA, and delivery of IV FCM. Restrictive transfusion protocols and lower transfusion trigger points are acknowledged as effective in reducing allogeneic blood transfusions [33], and multiple studies have affirmed the efficacy and safety of TXA in curbing blood loss [34,35]. One prospective study has also confirmed that the incidence of thromboembolic complications does not increase with the application of a TXA treatment [35]. Furthermore, prior evidence suggests that, in contrast to negative-pressure suction drains, neutral drainage or drain clamping may lower postoperative TBL through a tamponade effect [36,37]. In this context, the combined use of a cementless TKA under a PBM protocol emerges as a seemingly safe and efficient approach for patients with adequate bone quality. Nonetheless, further research investigating the effects of various PBM methods on post-TKA blood loss is necessary to provide corroboration.

The results of the present investigation have failed to establish an association between fixation method and greater blood loss after a PBM-aided TKA. In the logistic regression analysis, the prosthesis fixation method had no effect on the proportion of our patients with substantial Hb changes. Instead, the related factors were the preoperative Hb level and EBL volume during the acute postoperative period. We are, thus, in agreement with earlier publications implicating preoperative Hb levels and TXA delivery as potential risk factors for declines in Hb, as well as those similarly failing to link cementless TKA with increased bleeding risk [26,38]. It is unclear why some reports of exacerbated blood loss persist for cementless TKAs [9,39]. Yet, the heterogeneity of PBM protocols in use may explain this disparity. As mentioned earlier, a more comprehensive exploration of PBM protocols and their effects on hemodynamics may be a worthwhile pursuit.

There are several study limitations to address. First, our study was confined to an Asian population, primarily consisting of women (116/153, 75.8%). The underlying rea-sons for this apparent predilection for arthritis in Korean women are not yet understood [1]. Hence, extrapolating the observed results in terms of ethnicity and sex may be questioned. The considerable heterogeneity of PBM protocols among different institutions and surgeons may prohibit broader applicability as well. Another limitation arises from the bias introduced by the retrospective observational study design of the study, although we took steps to mitigate this bias through 1:1 PSM based on patient demographics. Furthermore, our sample size was predominantly determined for testing primary outcomes, so this analysis may have been underpowered and subject to type-II error in detecting all relevant outcomes. Since there were only 153 TKA patients in the present data, propensity score matching was performed only based on age, sex, and BMI, resulting in an even smaller number of 46 patients in each group with the possibility of an underpowered analysis of other possible confounding factors. Further prospective studies could be planned to eliminate these possible limitations. However, using a cementless TKA prosthesis has some considerations such as the optimal bone quality for cementless implant, suboptimal fixation, or fibrous bone apposition; so, for the patients’ safety, we will attempt a retrospective study using PSM. Additionally, all TKAs were performed by a single surgeon using a singe implant design that has the same configurational dimensions in both cemented and cementless TKAs. Additionally, there have been trials to find the safe indications for a recent cementless TKA prosthesis by using various methods including non-invasive radiologic tools [27]. An accumulation of these studies could have some possibilities to open the exact inclusion and exclusion criteria for cementless prostheses. If it is possible, further prospective randomized controlled trials to evaluate perioperative hemodynamic safety and efficacy between cemented and cementless prosthesis could be planned without worrying about patients’ safety. Lastly, it is worth noting that our evaluation of EBL levels was limited to postoperative Days 1 and 3, which, while informative, posed certain challenges to calculating blood loss. Among the various methods available, the equation developed by Lopez et al. demonstrated the closest agreement with directly measured values, albeit on the second postoperative day [21,40]. Despite these constraints, it is important to emphasize that this propensity score-matched study contributes a comprehensive analysis of postoperative hemodynamics and the identification of potential risk factors associated with increased blood loss following cementless TKAs, all conducted within the confines of a strict PBM protocol. The findings from this study offer valuable insights into the dynamics of blood loss in this context, which can further inform clinical practice and guide future research in the field of orthopedic surgery.

## 5. Conclusions

The present study has demonstrated that prosthesis fixation method (cementless vs. cemented) has no impact on post-TKA hemodynamics, including blood loss, Hb levels, and transfusion rates. In addition, our cementless TKA procedure under a PBM protocol showed no association with substantial blood loss.

## Figures and Tables

**Table 1:** Patient demographics and preoperative conditions before and after propensity score matching.

	Before Propensity Score Matching				After Propensity Score Matching			
	Cemented (n = 66)	Cementless (n = 87)	*p* Value	Stdiff	Cemented (n = 46)	Cementless (n = 46)	*p* Value	Stdiff
	Demographics *							
Age (year)	70.0 ± 6.7	66.5 ± 5.6	<0.01	0.574	69.0 ± 5.0	69.9 ± 4.8	0.399	0.184
Female †	52 (78.8)	64 (73.6)	0.455	0.177	39 (84.8)	38 (82.6)	0.778	0.088
Height (m)	1.54 ± 0.08	1.58 ± 0.07	<0.01	0.537	1.53 ± 0.07	1.56 ± 0.06	0.008	0.460
Weight (kg)	62.6 ± 7.9	67.0 ± 9.7	0.003	0.491	62.6 ± 7.5	65.0 ± 8.6	0.159	0.297
BMI (kg/m^2^)	26.5 ± 3.3	26.8 ± 3.2	0.625	0.093	26.9 ± 3.2	26.6 ± 3.5	0.714	0.090
	**Medical conditions †**							
ASA ≥ 3	6 (9.1)	12 (13.8)	0.371	0.259	5 (10.9)	8 (17.4)	0.369	0.301
Diabetes	12 (18.5)	22 (25.9)	0.282	0.232	5 (10.9)	13 (28.3)	0.036	0.647
Hypertension	35 (53.0)	54 (64.3)	0.164	0.205	25 (54.3)	31 (67.4)	0.200	0.304
Angina	5 (7.6)	4 (4.8)	0.483	0.293	4 (8.7)	1 (2.2)	0.168	0.802
Anti-platelet Tx.	12 (18.2)	12 (13.8)	0.460	0.181	8 (17.4)	8 (17.4)	1.000	0.000
Anti-coagulant Tx.	4 (6.1)	3 (3.4)	0.444	0.326	3 (6.5)	2 (4.3)	0.646	0.236
	Preoperative hematologic conditions *							
Hemoglobin (g/dL)	12.8 ± 1.2	13.2 ± 1.0	0.012	0.367	12.9 ± 1.2	13.1 ± 0.9	0.235	0.189
Hematocrit (%)	38.0 ± 3.6	39.3 ± 3.0	0.013	0.397	38.2 ± 3.6	38.8 ± 2.9	0.399	0.184
Preop PT (second)	11.3 ± 1.1	11.2 ± 0.7	0.563	0.112	11.3 ± 1.1	11.2 ± 0.6	0.513	0.113
Preop aPTT (second)	30.8 ± 2.5	31.3 ± 3.0	0.321	0.179	30.7 ± 2.5	30.8 ± 2.9	0.894	0.037
**Surgical conditions**								
Spinal anesthesia †	59 (89.4)	81 (93.1)	0.415	0.260	42 (91.3)	42 (91.3)	1.000	0.000
Torniquet time (minute) *	30.5 ± 8.4	24.8 ± 5.7	<0.01	0.815	29.7 ± 7.6	24.3 ± 6.9	<0.01	0.744

Stdiff: standardized mean difference; ASA: American Society of Anesthesiologists Classification; VTE: venous thromboembolism; PT: prothrombin time; aPTT: activated partial thromboplastin time. * Data are presented as means ± standard deviations. † Data are presented as numbers (percentage) of patients.

**Table 2:** Comparisons of hemodynamics and proportions of patients with hemoglobin drop > 3 g/dL between cemented and cementless TKA.

	Cemented (n = 46)	Cementless (n = 46)	*p* Value
Hemodynamics (mL) *			
Total blood loss	128.8 ± 93.5	116.7 ± 50.8	0.597
Total drain	99.4 ± 71.1	119.6 ± 103.3	0.289
Estimated blood loss			
PO 1 Day	611.5 ± 312.2	658.7 ± 266.0	0.437
PO 3 Day	1000.3 ± 425.0	1111.6 ± 392.3	0.195
Transfusion †	0 (0)	0 (0)	1.000
**Proportion of hemoglobin > 3 g/dL drop patient †**			
PO 1 Day	6 (13.0)	4 (8.7)	0.503
PO 3 Day	25 (54.3)	26 (57.8)	0.742
PO 1 Week	6 (19.4)	6 (20.0)	0.949
PO 2 Weeks	2 (7.4)	6 (15.8)	0.311
PO 6 Weeks	1 (4.8)	0 (0)	0.199

* Data are presented as means ± standard deviations. † Data are presented as numbers (percentage) of patients. PO: postoperative.

**Table 3:** Regression analysis for predictors of substantial Hb drop.

				Univariate Regression Analysis					
	Average Hb Drop (n = 69)	Substantial Hb Drop (n = 84)	*p* Value	B	SE	*p* Value	Adjusted ORs	95% CIs	
Demographics *									
Age	68.5 ± 6.9	67.6 ± 5.8	0.391	−0.022	0.026	0.389	0.978	0.929	1.029
Female †	55 (79.7)	61 (72.6)	0.308	−0.393	0.387	0.310	0.675	0.316	1.440
BMI	26.9 ± 3.3	26.4 ± 3.3	0.397	−0.043	0.051	0.395	0.958	0.868	1.058
**Medical conditions †**									
Diabetes	17 (24.6)	17 (20.2)	0.515	−0.253	0.394	0.515	0.776	0.361	1.666
Hypertension	35 (50.7)	54 (64.3)	0.091	0.559	0.331	0.092	1.749	0.913	3.348
Antiplatelet Tx.	8 (11.6)	16 (19.0)	0.207	0.585	0.468	0.211	1.794	0.718	4.486
Anticoagulant Tx.	1 (1.4)	6 (7.1)	0.094	1.655	1.093	0.130	5.231	0.614	44.540
ASA ≥ 3	5 (7.2)	13 (15.5)	0.116	0.852	0.554	0.124	2.344	0.792	6.938
**Surgical conditions**									
Cementless implant †	37 (53.6)	50 (59.5)	0.463	0.240	0.328	0.464	1.272	0.669	2.420
Spinal anesthesia †	60 (87.0)	80 (95.2)	0.068	1.099	0.625	0.079	3.000	0.882	10.207
Tourniquet time (min) *	27.0 ± 6.7	27.4 ± 8.2	0.720	0.008	0.022	0.719	1.008	0.966	1.052
Hemodynamics * (mL)									
Preoperative Hb (g/dL)	12.6 ± 1.0	13.4 ± 1.0	<0.01	0.855	0.191	<0.01	2.351	1.617	3.419
Total blood loss	110.0 ± 51.2	131.2 ± 86.2	0.196	0.005	0.004	0.205	1.005	0.998	1.012
Total drain	103.9 ± 77.9	128.9 ± 109.5	0.120	0.003	0.002	0.123	1.003	0.999	1.006
EBL PO 1 Day	464.5 ± 233.6	767.4 ± 264.8	<0.01	0.005	0.001	<0.01	1.005	1.003	1.007
EBL PO 3 Day	780.0 ± 449.7	1358.2 ± 309.1	<0.01	0.006	0.001	<0.01	1.006	1.004	1.008

Substantial Hb drop is defined as patients whose hemoglobin dropped by more than 3.0 g/dL on postoperative Day 3; Hb: hemoglobin; ASA: American Society of Anesthesiologists Classification; EBL: estimated blood loss; PO: postoperative. * Data are presented as means ± standard deviations. † Data are presented as numbers (percentage) of patients.

## Data Availability

Data will be provided by the corresponding author.
